# The Role of Neuropeptide Y mRNA Expression Level in Distinguishing Different Types of Depression

**DOI:** 10.3389/fnagi.2016.00323

**Published:** 2016-12-27

**Authors:** Yingying Yue, Haitang Jiang, Yingying Yin, Yuqun Zhang, Jinfeng Liang, Shenghua Li, Jun Wang, Jianxin Lu, Deqin Geng, Aiqin Wu, Yonggui Yuan

**Affiliations:** ^1^Department of Psychosomatics and Psychiatry, Institute of Psychosomatic Medicine, Zhongda Hospital, Medical School of Southeast UniversityNanjing, China; ^2^Department of Neurology, Jiangning Nanjing HospitalNanjing, China; ^3^Department of Neurology, The Affiliated Nanjing Hospital of Nanjing Medical UniversityNanjing, China; ^4^Department of Neurology, Gaochun County People’s HospitalNanjing, China; ^5^Department of Neurology, Affiliated Hospital of Xuzhou Medical CollegeXuzhou, China; ^6^Department of Psychosomatics, The First Affiliated Hospital of Suzhou UniversitySuzhou, China

**Keywords:** neuropeptide Y, protein, mRNA, stroke, depression

## Abstract

Previous studies demonstrate that the protein of neuropeptide Y (NPY) is abnormal in depression patients, but the changes of NPY in different types of depression are unclear. This study was aimed to examine protein and mRNA expression levels of NPY in 159 cases with four groups including post-stroke depression (PSD) group, stroke without depression (Non-PSD) group, major depressive disorder (MDD) group and normal control (NC) group. The protein and gene expression analysis were performed by enzyme-linked immunosorbent assay (ELISA) and quantitative polymerase chain reaction-based methods. One way analysis of variance (ANOVA), chi-square tests and nonparametric test were used to evaluate general characteristics, clinical and biological materials. In order to explore the role of NPY in different types of depression, the partial correlations, binary logistic regression analysis and receiver operating characteristic (ROC) curve were calculated for PSD and MDD groups. There are significant differences of NPY protein (*F*_df(3)_ = 5.167, *P* = 0.002) and mRNA expression levels ($\chi_{\text{Kruskal}}^{\text{2}}{}_{\text{-Wallis, df(3)}}$ = 20.541, *P* < 0.001) among four groups. Bonferroni multiple comparisons found that the NPY protein was significantly decreased in PSD (*F*_Bonferroni_ = −7.133, *P* = 0.002) and Non-PSD group (*F*_Bonferroni_ = −5.612, *P* = 0.018) compared with NC group. However, contrasted with MDD group, the mRNA expression was increased in PSD and Non-PSD group by nonparametric test (all *P* < 0.05). In binary logistic analyses, NPY mRNA expression was independent predictors of PSD (odds ratio: 1.452, 95% CI, 1.081–1.951, *P* = 0.013). The ROC curve showed NPY mRNA had a general prognostic accuracy (area under the curve: 0.766, 95% CI, 0.656–0.876, *P* < 0.001). This is the first study to explore the distinguishing function of NPY in different types of depression. It will provide help in the identification of different subtypes of depression.

## Introduction

Depression is one of the most frequently encountered forms of mental illness and more than 350 million people battle with it every day (Smith, 2014). Post-stroke depression (PSD) as a subtype of depression is a frequent complication after stroke, it is related to a variety of adverse health outcome including increased disability, difficult to recover and risk of suicide (Paolucci et al., 2001; Pompili et al., 2012). According to a meta-analysis, the prevalence of depression was 33% among stroke survivors by pooled estimate (Hackett et al., 2005).

Despite the high morbidity of PSD and major depressive disorder (MDD), there is yet little insight into the underlying neurobiological mechanisms. Preclinical and clinical studies suggest that many mechanisms are involved in this disorder including neurotrophic signaling, cellular plasticity and activation of the hypothalamic-pituitary-adrenal (HPA) axis (Spalletta et al., 2006; Loubinoux et al., 2012). In the aspect of the neurotrophic and neurochemical factors, brain-derived neurotrophic factor, glial cell line-derived neurotrophic factor and vascular endothelial growth factor were proved to play an important role in the pathophysiology of depression and antidepressant action of therapeutic interventions (Sharma et al., 2016). In addition, Neuropeptide Y (NPY), a 36-amino acid peptide, is widely distributed in the central and peripheral nervous systems such as most cortical areas, hippocampus, amygdala, olfactory bulb and spinal cord of the rat and human brain (Dumont et al., 1992). These brain regions are closely related to stress responses and mood disorders. With this in mind, researchers suggested that it as neuromodulators associated with MDD (Morales-Medina et al., 2010). NPY plays a regulatory role in feeding, body weight, sleep, cognition and emotion (Gehlert, 2004). In addition, NPY was found to regulate neurogenesis in the hippocampus. Significantly decreased NPY in the dorsal hippocampus was found in the maternally separated rats and Flinders sensitive line (FSL) rats (a genetic-based model of depression-like behavior; Caberlotto et al., 1999; Jiménez-Vasquez et al., 2001). Moreover, human and postmortem studies found decreased NPY levels in cerebrospinal fluid (CSF), plasma the frontal cortex and the caudate nucleus of depressive or attempted suicide patients, especially in repeatedly attempted suicides (Widerlöv et al., 1988; Widdowson et al., 1992; Westrin et al., 1999; Heilig, 2004). However, the NPY mRNA which was region specific in depressive-like animal models, significantly decreased in the nucleus accumbens, medial amygdala, hippocampal dentate gyrus, CA regions and prefrontal cortex, but increased in the arcuate nucleus and anterior cingulate cortex in the FSL and chronic mild stressed rats (Caberlotto et al., 1998; Sergeyev et al., 2005; Bjørnebekk et al., 2006; Zambello et al., 2008; Melas et al., 2012).

Withal NPY mediates neuroprotection against focal ischemia and regulates cerebral blood flow (Lundberg et al., 1982; Tuor et al., 1990; Cheung and Cechetto, 2000). Previous studies showed that NPY protein levels were increased in cortical and subcortical tissues around the lesions in the ischemic and hemorrhagic animal models (Kharlamov et al., 2007; Montaner et al., 2012). In plasma, NPY levels were significantly lower compared to the control group in the acute phase of female stroke patients (Baranowska et al., 2013).

Clinical and experimental investigations support that NPY is involved in the pathophysiology of depression and stroke. What is more, PSD as a disease integrated above two reciprocally relational factors, so we speculate NPY could be a molecular joint in this disease. Therefore, the first aim of this study was to explore the changes of NPY protein content and mRNA expression in PSD, stroke without depression (Non-PSD) and MDD patients. Then we further analyze the difference of NPY between the subtypes of depression including PSD and MDD patients.

## Materials and Methods

### Study Population

This study was approved by the Medical Ethics Committee for Clinical Research of Zhongda Hospital Affiliated to Southeast University. One-hundred and fifty-nine subjects including PSD (*n* = 39), Non-PSD (*n* = 42), MDD (*n* = 40) patients and normal control (NC; *n* = 38) were recruited during the period from July 2013 to December 2014 and they all gave written informed consent. Stroke patients were verified from computed tomography or magnetic resonance imaging reports within 7 days. MDD patients were carefully screened with the Structured Clinical Interview for the Diagnostic Statistical Manual of Mental Disorder (DSM-IV; SCID) by psychiatrists treating two participants PSD patients fulfilled the following diagnostic criteria: (1) had stroke before, or stroke occurs earlier than depressive symptoms; (2) had three or more depressive symptoms in nine symptoms of MDD in DSM-IV, at least one of the symptoms (1. depressed mood, 2. loss of interest or pleasure) is present; (3) the symptoms cause clinically significant distress or impairment in social, occupational or other important areas of functioning; (4) depressive symptoms lasting more than 1 week; and (5) free of other major psychiatric disorders, including schizophrenia, bipolar disorder, substance abuse (caffeine, nicotine and alcohol; Yue et al., 2015). Moreover, the patients are all antidepressants-naïve. The severity of depression and cognitive function were evaluated by Hamilton depression rating scale (HDRS-17) and mini-mental state examination (MMSE). HDRS was the most promising evaluation scale for PSD patients evidenced by a meta-analysis (Meader et al., 2014). MMSE as a global measurement of cognitive functioning was administered (Folstein et al., 1975).

### Blood Collection

Venous blood samples were collected via venipuncture in EDTA-anticoagulant and coagulant tubes after the admission. Whole blood was directly stored at −80°C for further study. Samples in coagulant tubes were separated by centrifugation (3000 rcf for 30 min at 4°C). The separated serum was stored at −80°C until the assay was performed.

### Determination of Serum Protein Level Using Enzyme-Linked Immunosorbent Assay (ELISA)

NPY levels were measured in duplicate using enzyme-linked immunosorbent assay (ELISA) method (Human NPY Quantikine kit, EMD Millipore Corporation, USA) following the manufacturer’s instructions. NPY content was expressed as pg of protein/ml of serum, with a detection limit sensitivity of 2 pg/ml.

### The Expression of NPY mRNA

#### RNA Extraction and Reverse Transcription

Total RNA was extracted from the peripheral blood lymphocytes using QIAamp RNA Blood Mini Kit (Qiagen, Hilden, Germany) following the manufacturer’s protocol. One microgram of total RNA got through the last process was reverse transcribed into cDNA using random hexanucleotide primers and Sensiscript Reverse Transcription Kit (Qiagen, Hilden, Germany) according to the instruction manual. Obtained cDNA was used in the quantitative polymerase chain reaction (qPCR).

#### Detection of Gene Expression Using qPCR Method

qPCR based on TaqMan technology was performed to test the NPY mRNA expression. Probe primers were designed using the Primer Express Software v2.0 by ABI company. β-actin (ACTB) was used as reference transcript and their primer sequences were listed in Table 1.

**Table 1:** **The primers for qPCR**.

Gene name	Primer	Sequence
NPY	NPY-taqman-F	5′-CGGAGGACATGGCCAGATAC-3′
	NPY-taqman-R	5′-GCCTGGTGATGAGGTTGATGTA-3′
	NPY-taqman	ACTCGGCGCTGCGA
ACTB	ACTB-F	5′-AGGCACCAGGGCGTGATG-3′
	ACTB-R	5′-CGCCCACATAGGAATCCTTCT-3′
	ACTB-Taqman	TGGGCATGGGT

*Note: qPCR, quantitative polymerase chain reaction; NPY, neuropeptide Y; ACTB, beta-actin*.

qPCR was carried out in a final volume of 16 μl, with 1 μl cDNA, 8 μl 2× SYBGEEN PCR mix, 1 μl of each primer and 5 μl H_2_O. Amplification was performed for 2 min at 95°C to activate FastStart Taq DNA polymerase and 40 rounds of 10 s at 94°C, 10 s at 59°C and 40 s at 72°C for amplification and signal analysis. ABI ViiA 7 Real-Time PCR System from Applied Biosystems was used to detect amplifications. Each sample was assayed in triplicate in independent reactions. Real-time PCR data were automatically calculated by the data analysis module. Relative expression changes were calculated with the 2^−∆∆^ method using ACTB as the reference transcript.

### Statistical Analysis

Demographic and clinical characteristics were described in quantitative terms of mean (M) and standard deviation (SD). Chi-square (*χ*^2^) test and one-way analysis of variance (ANOVA) were used to evaluate general characteristics. The HDRS and NYP protein were calculated by analysis of covariance (age and gender as covariates). The nonparametric test (Kruskal-Wallis H test) was used to analyze the mRNA level of NPY. The abnormal data determined by M ± 3 SD were excluded.

Then the following statistical analyses were calculated in the PSD and MDD groups. The partial correlation was used to evaluate bivariate correlations with age and gender as covariates. The influence of NPY in different types of depression was assessed by binary logistic regression analysis and the results were expressed as adjusted odds ratios (OR) with the corresponding 95% confidence interval (CI). Finally, receiver operating characteristic (ROC) curve was utilized to evaluate the accuracy of NPY to predict the PSD.

All analyses were conducted using SPSS Version 20.0 statistical software (SPSS Inc. Chicago, IL, USA).

## Results

### Demographic and Neuropsychological Results

The demographic and neuropsychological information for all participants are listed in Table 2. The age, gender, HDRS-17 and MMSE have significant differences (all *P* < 0.05) among four groups except the education level (mean square = 19.746, *F*_df(3)_ = 1.218, *P* = 0.305).

**Table 2:** **The demographic and clinical data among four groups**.

Item	PSD group (*n* = 39)	Non-PSD group (*n* = 42)	MDD group (*n* = 40)	NC group (*n* = 38)	*F*/*χ*^2^	*P* value
Age (years)	62.44 ± 10.34	61.10 ± 6.58	58.70 ± 9.92	57.58 ± 5.28	2.761	0.044^a^
Gender (male/female)	20/19	25/17	9/31	22/16	14.316	0.003^b^
Education level (years)	8.15 ± 4.36	9.43 ± 4.01	9.40 ± 4.77	8.24 ± 2.56	1.218	0.305^a^
HDRS	16.00 ± 5.45	3.36 ± 2.02	19.45 ± 4.75	2.18 ± 1.98	203.32	<0.001^c^
MMSE	22.64 ± 6.80	27.00 ± 2.45	27.08 ± 1.45	28.47 ± 1.45	28.46	<0.001^c^
NPY (pg/ml)^†^	17.65 ± 8.41^#^	19.17 ± 8.69^#^	20.50 ± 8.19	24.79 ± 7.93	5.167	0.002^a^
NPY mRNA	0.92 ± 1.27^*^	0.34 ± 0.34^*^	0.11 ± 0.16	1.00 ± 1.95	20.541	<0.001^d^
(2^−∆∆Ct^)^‡^

Note: ^a^one-way ANOVA. ^b^Chi-square test. ^c^Analysis of covariance, age, gender as covariates. ^d^Kruskal-Wallis H test. ^#^*P* < 0.05 compared with NC group. *P < 0.05 compared with MDD group. PSD, post-stroke depression; Non-PSD, stroke without depression; MDD, major depressive disorder; NC, normal control; HDRS, Hamilton depression rating scale, MMSE, Mini-mental state examination. ^†^Two abnormal data were excluded (two below the sensitive value in PSD group); ^‡^Fifteen abnormal data were excluded (three PSD patients, seven Non-PSD patients, two MDD patients and three NC subjects).

### The NPY Protein and mRNA Expression Level

In the protein level, analysis of covariance showed that there is a statistical difference among four groups (mean square = 357.279, *F*_df(3)_ = 5.167, *P* = 0.002), with two patients in PSD group, eliminated on account of being below the sensitivity. Compared with NC group, a significantly decreased content in PSD (*F*_Bonferroni_ = −7.133, standard error (SE) = 1.920, *P* = 0.002) and Non-PSD group (*F*_Bonferroni_ = −5.612, SE = 1.862, *P* = 0.018) was discovered by Bonferroni multiple comparisons (see Table 2).

In the mRNA expression aspect, the statistically significant difference was also found among four groups ($\chi_{\text{Kruskal}}^{\text{2}}{}_{\text{-Wallis, df(3)}}$ = 20.541, *P* < 0.001), however, multiple comparison tests showed that both the PSD and Non-PSD groups have significantly increased expression compared to MDD group (0.92 ± 1.27 in PSD vs. 0.11 ± 0.16 in MDD, 0.34 ± 0.34 in Non-PSD vs. 0.11 ± 0.16 in MDD, *all P* < 0.05, see Table 2).

### The Predictive Effect of NPY on PSD and MDD

**Figure 1:**
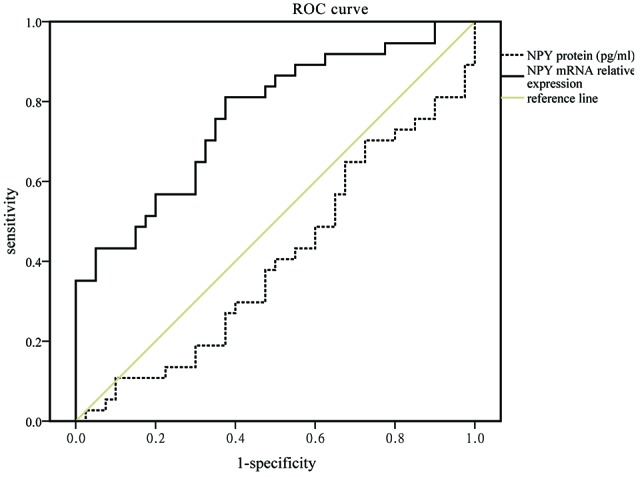
**Receiver operating characteristic (ROC) curve for mRNA expression and serum concentration of neuropeptide Y (NPY) which areas under the line were 0.766 (95% CI, 0.656–0.876, *P* < 0.001, solid black line) and 0.407 (95% CI, 0.275–0.540, *P* = 0.176, dotted black line), respectively**.

No relationship between the NPY and neuropsychological scales including HDRS-17 and MMSE was found in this study. In logistic analysis, serum NPY concentration and mRNA expression were independent predictors of PSD with OR of 0.933 (95% CI, 0.871–1.000; *P* = 0.050), 1.452 (95% CI, 1.081–1.951; *P* = 0.013), respectively (see Table 3). As showed in Figure 1, NPY mRNA had a higher prognostic accuracy (area under the curve (AUC), 0.766 (95% CI, 0.656–0.876), *P* < 0.001) as compared to serum concentration (AUC, 0.407 (95% CI, 0.275–0.540), *P* = 0.176).

**Table 3:** **The results of the logistic regression model in post-stroke depression (PSD) and major depressive disorder (MDD) groups**.

	β value	Standard error	*Warld* *χ*^2^ (df = 1)	*P* value	OR	95% CI
NPY protein (pg/ml)	−0.069	0.035	3.858	0.050	0.933	0.871–1.000
NPY-mRNA expression	0.373	0.151	6.137	0.013	1.452	1.081–1.951

*Note: The forward logistic regression analysis was used to determine the predictive value of NPY. NPY, neuropeptide Y; OR, odds ratios; CI, confidence intervals; Statistical significance was set at *P* < 0.05*.

## Discussion

The present study provides evidence that the protein and transcriptional changes of NPY occur in PSD and different depressions. The protein content shows a consistent decline in PSD, Non-PSD and MDD group, but only PSD and Non-PSD groups reach statistical significance when compared with NC group. While the transcriptional levels display significant differences between PSD, Non-PSD and MDD group. Moreover, the NPY mRNA expression can identify the different types of depression to some extent. It’s important to note that this is an exploratory study with small sample size, so replication of these findings in larger cohorts will be necessary for validation.

The reduced protein and mRNA of NPY in MDD group were obtained in genetic rat models of depression and other clinical research, suggesting that these changes most likely are triggered by stress (Lachman et al., 1992; Westrin et al., 1999; Melas et al., 2013). Previous studies demonstrated that enhancing NPY function boosts resilience (Christiansen et al., 2011; Southwick and Charney, 2012; Cohen et al., 2015). NPY can be served as a potential anti-depressive factor and used to monitor treatment of depression by recent findings (Bigio et al., 2016; Nakhate et al., 2016; Ozsoy et al., 2016; Wagner et al., 2016). NPY is one of the most abundant peptides in the central nervous system and mediates mood disorder, stress response and antidepressant effect mainly through the Y1 and Y2 receptors, which are densely expressed in the cortex, hippocampus and amygdala (Dumont et al., 1998; Price and Drevets, 2012). So it may be speculated that NPY participates in MDD through modulating the internal and mutual function of these brain regions. Furthermore, Mickey et al. (2011) found that individuals with low-expression NPY genotypes were inclined to negative affective experience under stress. Taken together, the evidence suggested that low NPY increased the risk for depression through adding sensitivity to negative stimuli and declining brain reserve capacity at the cellular levels and also possibly at the neural circuit levels (Freret et al., 2015).

Compared with the NC group, the change of protein had a significant difference in PSD and Non-PSD patients. The possible explanation could be that stress was the cause for stroke and depression (Sergeyev et al., 2005). Another mechanism could be HPA axis activation, then subsequent suppression of NPY levels by elevating glucocorticoid (Holsboer, 2000; Makino et al., 2000). Indeed, the neuroprotection role of NPY suggests that the reduced level counteracts the positive influence on the central and peripheral systems in PSD diseases. Other mechanisms including serotonin receptors and exaggerated inflammatory cytokines initiated by brain injury and persistent neuronal death might contribute to PSD (Popa-Wagner et al., 2014; Buga et al., 2016).

However, the result of mRNA level has a significant difference between PSD and MDD groups. It was consistent with the results of ROC and logistic regression analyses, which showed that NPY mRNA could be conducive to distinguish different subtypes of depression (PSD and MDD; AUC = 0.766). These suggest that PSD process the discrepantly pathological mechanisms though a subtype of depression encompasses many overlapping syndromes similar to MDD (Alexopoulos, 2003). The possible explanation was that the gene-expression changes were effected by the stress property (acute or chronic) as well as the time accumulation (Conrad and McEwen, 2000; Yang et al., 2013). MDD patients had decreased NPY protein and mRNA caused by chronic stress, while PSD patients mixed acute stress of stroke which leads to a transitory rise of mRNA, but the protein had no time to be affected for simultaneously collected blood samples. Moreover, the selected analyte of NPY mRNA expression, potentially provide new insights into exploring the molecular mechanism of different subtypes of depression.

Several issues should be considered in this exploring study. First, the present study yielded a relatively small sample size, so the findings cannot be considered categorical. Second, this was a cross-sectional study and the serum levels and mRNA of NPY measured at the acute stage of stroke patients, hence, the long-term dynamic evolution process of these biological characters is not known. Third, the NPY might be influenced by other neurochemical or genetic material, so it is important to enroll a set of subjects with high homogeneity. Given these limitations, the results should be considered preliminary and future studies should be well designed with several time points of large number of participants.

In conclusion, the present study demonstrates that NPY is abnormal in stroke, PSD and MDD patients and it has a significant distinguished function in the different subtypes of depression. These findings, together with previously published results, suggest that NPY system provides a novel version for the mechanism of depression pathology.

## Author Contributions

YYuan designed the study. YYue, HJ, YYin, YZ, JLiang, SL, JW, JLu and DG collected the participants. YYue experimented and analyzed the data, then wrote the article. AW, YYuan, YYue and all authors reviewed and approved for publication.

## Conflict of Interest Statement

The authors declare that the research was conducted in the absence of any commercial or financial relationships that could be construed as a potential conflict of interest.

## References

[B1] Alexopoulos G. S. (2003). Vascular disease, depression, and dementia. J. Am. Geriatr. Soc. 51, 1178–1180. 10.1046/j.1532-5415.2003.51373.x 12890087

[B2] Baranowska B. Kochanowski J. Grudniak M. Wolinska-Witort E. Kalisz M. Bik W. (2013). Plasma NPY concentrations in women with acute ischemic stroke. Neuro Endocrinol. Lett. 34, 124–128. 23645309

[B3] Bigio B. Mathé A. A. Sousa V. C. Zelli D. Svenningsson P. McEwen B. S.. (2016). Epigenetics and energetics in ventral hippocampus mediate rapid antidepressant action: implications for treatment resistance. Proc. Natl. Acad. Sci. U S A 113, 7906–7911. 10.1073/pnas.1603111113 27354525 PMC4948346

[B4] Bjørnebekk A. Mathé A. A. Brené S. (2006). Running has differential effects on NPY, opiates, and cell proliferation in an animal model of depression and controls. Neuropsychopharmacology 31, 256–264. 10.1038/sj.npp.1300820 16034445

[B5] Buga A. M. Ciobanu O. Bădescu G. M. Bogdan C. Weston R. Slevin M.. (2016). Up-regulation of serotonin receptor 2B mRNA and protein in the peri-infarcted area of aged rats and stroke patients. Oncotarget 7, 17415–17430. 10.18632/oncotarget.8277 27013593 PMC4951222

[B6] Caberlotto L. Fuxe K. Overstreet D. H. Gerrard P. Hurd Y. L. (1998). Alterations in neuropeptide Y and Y1 receptor mRNA expression in brains from an animal model of depression: region specific adaptation after fluoxetine treatment. Mol. Brain Res. 59, 58–65. 10.1016/s0169-328x(98)00137-5 9729278

[B7] Caberlotto L. Jimenez P. Overstreet D. H. Hurd Y. L. Mathé A. A. Fuxe K. (1999). Alterations in neuropeptide Y levels and Y1 binding sites in the Flinders sensitive line rats, a genetic animal model of depression. Neurosci. Lett. 265, 191–194. 10.1016/s0304-3940(99)00234-7 10327163

[B8] Cheung R. T. Cechetto D. F. (2000). Neuropeptide Y-Y1 receptor antisense oligodeoxynucleotide increases the infarct volume after middle cerebral artery occlusion in rats. Neuroscience 98, 771–777. 10.1016/s0306-4522(00)00159-7 10891620

[B9] Christiansen S. H. Olesen M. V. Wörtwein G. Woldbye D. P. (2011). Fluoxetine reverts chronic restraint stress-induced depression-like behaviour and increases neuropeptide Y and galanin expression in mice. Behav. Brain Res. 216, 585–591. 10.1016/j.bbr.2010.08.044 20816900

[B10] Cohen S. Vainer E. Matar M. A. Kozlovsky N. Kaplan Z. Zohar J.. (2015). Diurnal fluctuations in HPA and neuropeptide Y-ergic systems underlie differences in vulnerability to traumatic stress responses at different zeitgeber times. Neuropsychopharmacology 40, 774–790. 10.1038/npp.2014.257 25241802 PMC4289967

[B11] Conrad C. D. McEwen B. S. (2000). Acute stress increases neuropeptide Y mRNA within the arcuate nucleus and hilus of the dentate gyrus. Mol. Brain Res. 79, 102–109. 10.1016/s0169-328x(00)00105-4 10925147

[B12] Dumont Y. Jacques D. Bouchard P. Quirion R. (1998). Species differences in the expression and distribution of the neuropeptide Y Y1, Y2, Y4, and Y5 receptors in rodents, guinea pig, and primates brains. J. Comp. Neurol. 402, 372–384. 10.1002/(SICI)1096-9861(19981221)402:3<::AID-CNE6>3.3.CO;2-U 9853905

[B13] Dumont Y. Martel J. C. Fournier A. St-Pierre S. Quirion R. (1992). Neuropeptide Y and neuropeptide Y receptor subtypes in brain and peripheral tissues. Prog. Neurobiol. 38, 125–167. 10.1016/0301-0082(92)90038-g 1312243

[B14] Folstein M. F. Folstein S. E. McHugh P. R. (1975). “Mini-mental state”. A practical method for grading the cognitive state of patients for the clinician. J. Psychiatr. Res. 12, 189–198. 10.1016/0022-3956(75)90026-6 1202204

[B15] Freret T. Gaudreau P. Schumann-Bard P. Billard J. M. Popa-Wagner A. (2015). Mechanisms underlying the neuroprotective effect of brain reserve against late life depression. J. Neural Transm. (Vienna) 122, S55–S61. 10.1007/s00702-013-1154-2 24390152

[B16] Gehlert D. R. (2004). Introduction to the reviews on neuropeptide Y. Neuropeptides 38, 135–140. 10.1016/j.npep.2004.07.002 15337366

[B17] Hackett M. L. Yapa C. Parag V. Anderson C. S. (2005). Frequency of depression after stroke: a systematic review of observational studies. Stroke 36, 1330–1340. 10.1161/01.STR.0000165928.19135.35 15879342

[B18] Heilig M. (2004). The NPY system in stress, anxiety and depression. Neuropeptides 38, 213–224. 10.1016/j.npep.2004.05.002 15337373

[B19] Holsboer F. (2000). The corticosteroid receptor hypothesis of depression. Neuropsychopharmacology 23, 477–501. 10.1016/s0893-133x(00)00159-7 11027914

[B20] Jiménez-Vasquez P. A. Mathé A. A. Thomas J. D. Riley E. P. Ehlers C. L. (2001). Early maternal separation alters neuropeptide Y concentrations in selected brain regions in adult rats. Dev. Brain Res. 131, 149–152. 10.1016/s0165-3806(01)00264-4 11718845

[B21] Kharlamov E. A. Kharlamov A. Kelly K. M. (2007). Changes in neuropeptide Y protein expression following photothrombotic brain infarction and epileptogenesis. Brain Res. 1127, 151–162. 10.1016/j.brainres.2006.09.107 17123484 PMC1802128

[B22] Lachman H. M. Papolos D. F. Weiner E. D. Ramazankhana R. Hartnick C. Edwards E.. (1992). Hippocampal neuropeptide Y mRNA is reduced in a strain of learned helpless resistant rats. Mol. Brain Res. 14, 94–100. 10.1016/0169-328x(92)90015-4 1353857

[B23] Loubinoux I. Kronenberg G. Endres M. Schumann-Bard P. Freret T. Filipkowski R. K.. (2012). Post-stroke depression: mechanisms, translation and therapy. J. Cell. Mol. Med. 16, 1961–1969. 10.1111/j.1582-4934.2012.01555.x 22348642 PMC3822966

[B24] Lundberg J. M. Terenius L. Hökfelt T. Martling C. R. Tatemoto K. Mutt V.. (1982). Neuropeptide Y (NPY)-like immunoreactivity in peripheral noradrenergic neurons and effects of NPY on sympathetic function. Acta Physiol. Scand. 116, 477–480. 10.1111/j.1748-1716.1982.tb07171.x 6763452

[B25] Makino S. Baker R. A. Smith M. A. Gold P. W. (2000). Differential regulation of neuropeptide Y mRNA expression in the arcuate nucleus and locus coeruleus by stress and antidepressants. J. Neuroendocrinol. 12, 387–395. 10.1046/j.1365-2826.2000.00451.x 10792576

[B26] Meader N. Moe-Byrne T. Llewellyn A. Mitchell A. J. (2014). Screening for poststroke major depression: a meta-analysis of diagnostic validity studies. J. Neurol. Neurosurg. Psychiatry 85, 198–206. 10.1136/jnnp-2012-304194 23385849

[B27] Melas P. A. Lennartsson A. Vakifahmetoglu-Norberg H. Wei Y. Åberg E. Werme M.. (2013). Allele-specific programming of Npy and epigenetic effects of physical activity in a genetic model of depression. Transl. Psychiatry 3:e255. 10.1038/tp.2013.31 23652932 PMC3669918

[B28] Melas P. A. Mannervik M. Mathe A. A. Lavebratt C. (2012). Neuropeptide Y: identification of a novel rat mRNA splice-variant that is downregulated in the hippocampus and the prefrontal cortex of a depression-like model. Peptides 35, 49–55. 10.1016/j.peptides.2012.02.020 22406386

[B29] Mickey B. J. Zhou Z. Heitzeg M. M. Heinz E. Hodgkinson C. A. Hsu D. T.. (2011). Emotion processing, major depression and functional genetic variation of neuropeptide Y. Arch. Gen. Psychiatry 68, 158–166. 10.1001/archgenpsychiatry.2010.197 21300944 PMC3091621

[B30] Montaner J. Mendioroz M. Delgado P. Garcia-Berrocoso T. Giralt D. Merino C.. (2012). Differentiating ischemic from hemorrhagic stroke using plasma biomarkers: the S100B/RAGE pathway. J. Proteomics 75, 4758–4765. 10.1016/j.jprot.2012.01.033 22343074

[B31] Morales-Medina J. C. Dumont Y. Quirion R. (2010). A possible role of neuropeptide Y in depression and stress. Brain Res. 1314, 194–205. 10.1016/j.brainres.2009.09.077 19782662

[B32] Nakhate K. T. Yedke S. U. Bharne A. P. Subhedar N. K. Kokare D. M. (2016). Evidence for the involvement of neuropeptide Y in the antidepressant effect of imipramine in type 2 diabetes. Brain Res. 1646, 1–11. 10.1016/j.brainres.2016.05.035 27208493

[B33] Ozsoy S. Olguner Eker O. Abdulrezzak U. (2016). The effects of antidepressants on neuropeptide Y in patients with depression and anxiety. Pharmacopsychiatry 49, 26–31. 10.1055/s-0035-1565241 26789271

[B34] Paolucci S. Antonucci G. Grasso M. G. Morelli D. Troisi E. Coiro P.. (2001). Post-stroke depression, antidepressant treatment and rehabilitation results. A case-control study. Cerebrovasc. Dis. 12, 264–271. 10.1159/000047714 11641594

[B35] Pompili M. Venturini P. Campi S. Seretti M. E. Montebovi F. Lamis D. A.. (2012). Do stroke patients have an increased risk of developing suicidal ideation or dying by suicide? An overview of the current literature. CNS Neurosci. Ther. 18, 711–721. 10.1111/j.1755-5949.2012.00364.x 22943140 PMC6493438

[B36] Popa-Wagner A. Buga A. M. Tica A. A. Albu C. V. (2014). Perfusion deficits, inflammation and aging precipitate depressive behaviour. Biogerontology 15, 439–448. 10.1007/s10522-014-9516-1 25033986

[B37] Price J. L. Drevets W. C. (2012). Neural circuits underlying the pathophysiology of mood disorders. Trends Cogn. Sci. 16, 61–71. 10.1016/j.tics.2011.12.011 22197477

[B38] Sergeyev V. Fetissov S. Mathé A. A. Jimenez P. A. Bartfai T. Mortas P.. (2005). Neuropeptide expression in rats exposed to chronic mild stresses. Psychopharmacology (Berl) 178, 115–124. 10.1007/s00213-004-2015-3 15719227

[B39] Sharma A. N. da Costa e Silva B. F. B. Soares J. C. Carvalho A. F. Quevedo J. (2016). Role of trophic factors GDNF, IGF-1 and VEGF in major depressive disorder: a comprehensive review of human studies. J. Affect. Disord. 197, 9–20. 10.1016/j.jad.2016.02.067 26956384 PMC4837031

[B40] Smith K. (2014). Mental health: a world of depression. Nature 515, 181. 10.1038/515180a 25391942

[B41] Southwick S. M. Charney D. S. (2012). The science of resilience: implications for the prevention and treatment of depression. Science 338, 79–82. 10.1126/science.1222942 23042887

[B42] Spalletta G. Bossù P. Ciaramella A. Bria P. Caltagirone C. Robinson R. G. (2006). The etiology of poststroke depression: a review of the literature and a new hypothe-sis involving inflammatory cytokines. Mol. Psychiatry 11, 984–991. 10.1038/sj.mp.4001879 16894392

[B43] Tuor U. I. Kelly P. A. Edvinsson L. McCulloch J. (1990). Neuropeptide Y and the cerebral circulation. J. Cereb. Blood Flow Metab. 10, 591–601. 10.1038/jcbfm.1990.110 2384533

[B44] Wagner L. Kaestner F. Wolf R. Stiller H. Heiser U. Manhart S.. (2016). Identifying neuropeptide Y (NPY) as the main stress-related substrate of dipeptidyl peptidase 4 (DPP4) in blood circulation. Neuropeptides 57, 21–34. 10.1016/j.npep.2016.02.007 26988064

[B45] Westrin A. Ekman R. Träskman-Bendz L. (1999). Alterations of corticotropin releasing hormone (CRH) and neuropeptide Y (NPY) plasma levels in mood disorder patients with a recent suicide attempt. Eur. Neuropsychopharmacol. 9, 205–211. 10.1016/s0924-977x(98)00026-1 10208289

[B46] Widdowson P. S. Ordway G. A. Halaris A. E. (1992). Reduced neuropeptide Y concentrations in suicide brain. J. Neurochem. 59, 73–80. 10.1111/j.1471-4159.1992.tb08877.x 1613514

[B47] Widerlöv E. Lindström L. H. Wahlestedt C. Ekman R. (1988). Neuropeptide Y and peptide YY as possible cerebrospinal fluid markers for major depression and schizophrenia, respectively. J. Psychiatr. Res. 22, 69–79. 10.1016/0022-3956(88)90030-1 3397912

[B48] Yang Y. Zhang J. Liu H. Wang J. Xin J. Deng M. (2013). Changes in levels of hypoxia-induced mediators in rat hippocampus during chronic cerebral hypoperfusion. Neurochem. Res. 38, 2433–2439. 10.1007/s11064-013-1158-1 24072673

[B49] Yue Y. Liu R. Lu J. Wang X. Zhang S. Wu A.. (2015). Reliability and validity of a new post-stroke depression scale in Chinese population. J. Affect. Disord. 174, 317–323. 10.1016/j.jad.2014.11.031 25528001

[B50] Zambello E. Jiménez-Vasquez P. A. El Khoury A. Mathé A. A. Caberlotto L. (2008). Acute stress differentially affects corticotropin-releasing hormone mRNA expression in the central amygdala of the “depressed” flinders sensitive line and the control flinders resistant line rats. Prog. Neuropsychopharmacol. Biol. Psychiatry 32, 651–661. 10.1016/j.pnpbp.2007.11.008 18077069

